# Radiographic Findings of Evolving Sequelae of Cerebellar, Hippocampal, and Basal Nuclei Transient Edema With Restricted Diffusion (CHANTER) Syndrome in a 37-Year-Old Patient

**DOI:** 10.7759/cureus.73467

**Published:** 2024-11-11

**Authors:** Brady A Wahlstrom, Ian Matthews, Cameron Brown, Shamseldeen Y Mahmoud

**Affiliations:** 1 Medicine, Saint Louis University School of Medicine, Saint Louis, USA; 2 Radiology, Saint Louis University School of Medicine, Saint Louis, USA

**Keywords:** chanter syndrome, hippocampal restricted diffusion, mri, opiods, opioid-induced amnesia

## Abstract

Cerebellar, Hippocampal, and Basal Nuclei Transient Edema with Restricted Diffusion (CHANTER) syndrome is a recently recognized distinct clinicoradiographic pattern of neurologic injury occurring most commonly following polysubstance or opioid abuse. Patients present acutely with unresponsiveness or coma. Magnetic resonance (MR) imaging demonstrates key findings, including bilateral diffusion restriction in the cerebellar cortices and hippocampi and variable diffusion restriction in the basal ganglia. Additionally, cerebellar cytotoxic edema can exert a mass effect on the adjacent fourth ventricle, causing obstructive hydrocephalus and requiring emergent intervention to prevent brain herniation and death. Here, we present a 37-year-old male patient who arrived at the Emergency Department with non-responsiveness in the setting of positive toxicology for cannabis, cocaine, and fentanyl. One day following the presentation, an MRI demonstrated restricted diffusion of the bilateral cerebellar hemispheres and bilateral hippocampi and small foci of restricted diffusion involving the basal ganglia. T2 fluid-attenuated inversion recovery (T2 FLAIR) hyperintensities involving the bilateral precentral gyri were also identified. Additional diagnostic workup, including electroencephalogram (EEG), vessel imaging, and viral panels, effectively ruled out alternative diagnoses, making CHANTER most likely. Following medical treatment without surgical intervention, the patient was discharged to an acute rehab facility with symptoms of impaired judgment, difficulty following commands, and difficulty walking. In the following months, the patient improved in cognitive function and gait. MRI at three months demonstrated interval resolution of previous T2 FLAIR hyperintensities, restricted diffusion zones, and development of new T2 FLAIR hyperintensities in the periventricular and subcortical white matter. This case highlights the need for prompt recognition of radiographic features of CHANTER, as affected patients may demonstrate significant recovery of neurologic status in the months following injury, compared to other patterns of hypoxic brain injury. These patients, therefore, merit more aggressive treatment to maximize recovery.

## Introduction

Cerebellar, hippocampal, and basal nuclei transient edema with restricted diffusion (CHANTER) syndrome is a constellation of imaging and clinical findings first categorized in 2019 [1]. The clinical presentations include altered mental status or coma after recent ingestion of drugs of abuse paired with radiographic evidence of cytotoxic edema of the bilateral cerebellum, hippocampi, and basal ganglia. There may also be obstructive hydrocephalus secondary to associated edema in the posterior fossa [2-4]. This syndrome has been chiefly characterized alongside drug abuse, most commonly opioid abuse, though it has been described with other substances as well and has been seen in patients as young as two years old [5]. This syndrome has been associated with poor outcomes, particularly if hydrocephalus is present [2,6]. However, there have been reports of near-full recoveries with treatment [7]. As we continue to fight the ongoing opioid epidemic, it is essential to recognize this potentially devastating syndrome early for the best possible outcomes. Over the last few years, CHANTER syndrome has become more recognized in the literature as numbers of opioid-related hospitalizations continue to rise with the increased use of street fentanyl. However, there is still scarce data on this new entity. We aim to add to the growing pool of data and images regarding CHANTER syndrome to help radiologists and clinicians better recognize this syndrome and keep it in their differential diagnoses.

## Case presentation

Our patient is a 37-year-old male patient with a history of heavy cocaine, alcohol, and cannabis use who was found unresponsive in his car. Emergency medical services (EMS) brought him to the emergency room (ED). En route, EMS noted pinpoint pupils and administered a 4 mg dose of Narcan®. Upon arrival, the patient was nonresponsive and only withdrew to noxious solid stimuli. He was intubated for airway protection. However, other vital signs were otherwise within normal limits and stable. The patient was admitted to the ICU, where his drug screen was positive for cocaine, cannabis, and fentanyl, though fentanyl had been given in the ED before the screen. 

**Figure 1:**
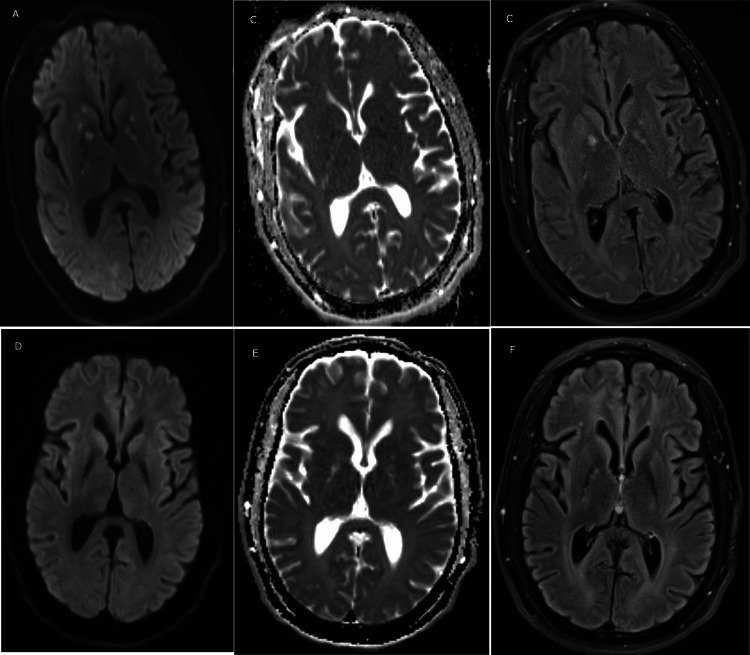
MRI images of the bilateral basal ganglia at presentation and at the three-month follow-up ADC/DWI images show diffusion restriction and T2 FLAIR shows edema of the bilateral basal ganglia at presentation and at the three-month follow-up A. Diffusion-weighted imaging (DWI) image at presentation; B. Apparent diffusion coefficient (ADC) image at presentation; C. T2 fluid-attenuated inversion recovery (FLAIR) image at presentation; D. DWI image at three months; E. ADC image at three months; F. T2 FLAIR image at three months.

**Figure 2:**
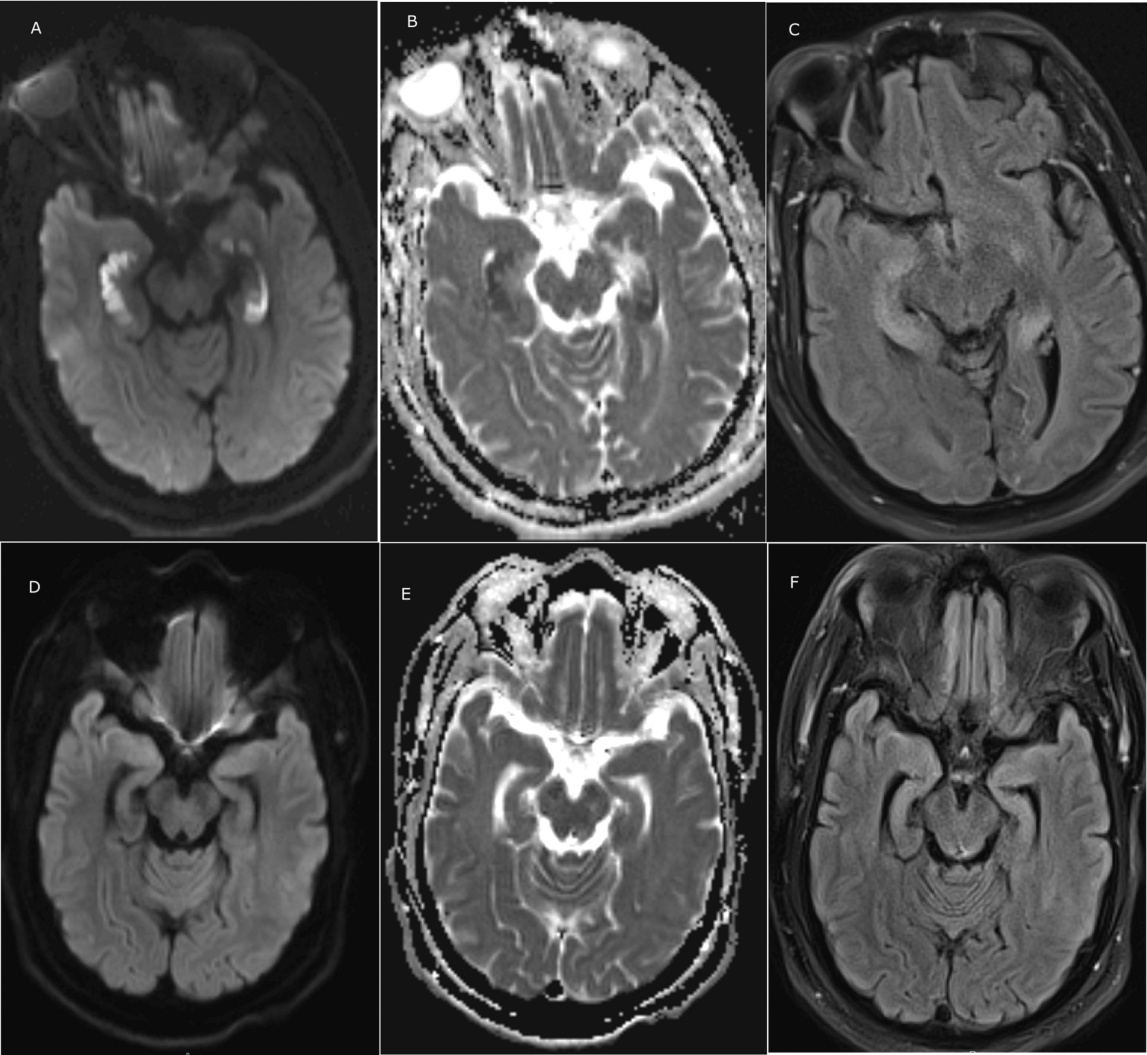
MRI images of the bilateral hippocampi at presentation and at the three-month follow-up ADC/DWI images show diffusion restriction and T2 FLAIR shows edema of the bilateral hippocampi at presentation and at the three-month follow-up A. Diffusion-weighted imaging (DWI) image at presentation; B. Apparent diffusion coefficient (ADC) image at presentation; C. T2 fluid-attenuated inversion recovery (FLAIR) image at presentation; D. DWI image at three months; E. ADC image at three months; F. T2 FLAIR image at three months.

**Figure 3:**
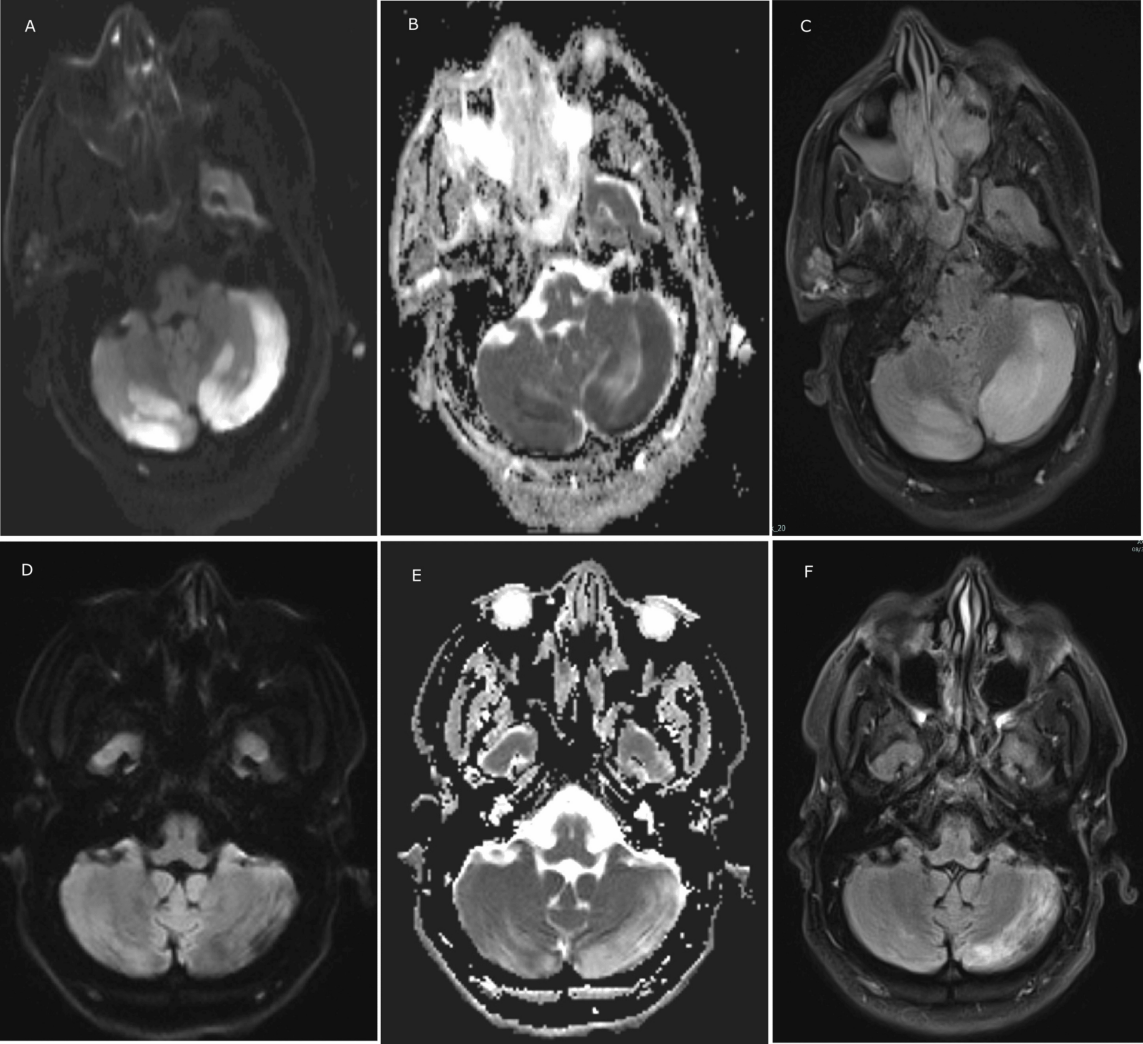
MRI images of the bilateral cerebellum at presentation and at the three-month follow-up ADC/DWI images show diffusion restriction and T2 FLAIR images show edema of the bilateral cerebellum at presentation and at the three-month follow-up A. Diffusion-weighted imaging (DWI) image at presentation; B. Apparent diffusion coefficient (ADC) image at presentation; C. T2 fluid-attenuated inversion recovery (FLAIR) image at presentation; D. DWI image at three months; E. ADC image at three months; F. T2 FLAIR image at three months.

A continuous electroencephalogram (EEG) was started, showing no seizure activity but generalized slowing. A Magnetic Resonance Image (MRI) of the brain with and without contrast showed scattered areas of restricted diffusion in the bilateral cerebellum, hippocampi, and basal ganglia (Figures 1-3). The following day, the patient’s condition worsened, so a Computed Tomography (CT) scan of the head was obtained, showing no signs of hydrocephalus or hemorrhage.

After three days, the patient was successfully extubated and weaned off sedation. Over the next 10 days, he began to regain awareness slowly, though his hospital course was further complicated by acute kidney injury and aspiration pneumonia, for which he received antibiotics. The patient was discharged to an acute rehab facility after a hospital stay of 22 days with continued symptoms of impaired judgment and difficulty following simple commands without significant coaching, as well as an inability to ambulate. Near discharge, the patient had a St. Louis University Mental Status (SLUMS) cognitive assessment score of 6/30, indicating significant cognitive deficiencies. 

**Figure 4:**
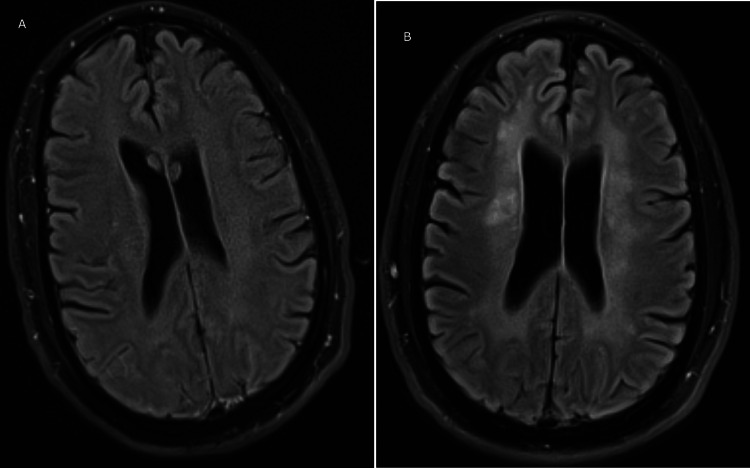
T2 FLAIR images from patient presentation and three-month follow up T2 FLAIR images of the patient at presentation and at the three-month follow-up showing evolving periventricular and deep subcortical white matter hyperintensities. A. Image at presentation; B. Image at three months.

The patient showed marked improvement over the next three months from his state at discharge. However, he reported persistent short-term memory problems and occasional times when he forgot who he was for brief stretches, and he could more easily follow commands. Additionally, he was able to transition from using a walker to using a cane or occasionally walking unassisted. A neurologic exam at this time showed intact cranial nerves, baseline motor function and reflexes, and no sensory abnormalities. No cerebellar signs were present, including a negative Romberg sign, and the patient was able to perform a tandem walk unassisted. A three-month follow-up MRI showed resolution of the previously seen restriction diffusion of the bilateral cerebellum, basal ganglia, and hippocampi (Figures 1-3). It also showed newly developed T2 FLAIR Hyperintensities in the periventricular and subcortical white matter that was not present on the initial scan (Figure 4).

## Discussion

CHANTER syndrome is still a new entity that is relatively unknown to many clinicians and radiologists alike. Given that the lesions are bilateral and do not follow typical vascular territories, and there is usually minimal cortical involvement, the diffusion restriction shown on the MRI should not be mistaken for infarction [1,3]. The main differentials include other conditions, such as heroin-associated spongiform leukoencephalopathy, Posterior reversible encephalopathy syndrome, and opioid-associated amnestic syndrome [3,8]. More research must be done to determine if these diseases and pediatric opioid use-associated neurotoxicity with cerebellar edema (POUNCE) are all part of a spectrum of the same pathology [3].

Given the association this syndrome has with drug abuse, the clinical history is key to making this diagnosis with imaging, but it is believed that the imaging findings for CHANTER syndrome are distinct enough that radiologists will often be the first to consider this diagnosis and should mention its possibility whenever suspected to aid in rapid diagnosis and treatment [3]. Without fast treatment, catastrophic deficits or death may result from obstructive hydrocephalus or herniation.

It is thought that the imaging findings in CHANTER syndrome may be due to a combination of higher concentrations of opioid receptors and the higher sensitivity to hypoxic injury of the cerebellum, hippocampi, and basal ganglia [3,9]. However, this may not be the complete picture, as it has been seen with exposure to other nonopioid substances as well [1].

This patient showed marked recovery of gait and cognition compared to his initial presentation, and his imaging showed resolved diffusion restriction and edema. However, he remains with significant deficits in memory and limited mobility. This case helps illustrate just how variable the outcomes of this syndrome are. More cases need to be documented and analyzed to understand better CHANTER syndrome, its pathophysiology, and its long-term sequalae.

## Conclusions

CHANTER syndrome is newly recognized and not fully understood as the complete pathophysiology has yet to be fully uncovered. Without timely opioid reversal, cardiovascular and respiratory support, and close neurological monitoring for seizures or signs of intracranial mass effect, it can have devastating, long-lasting outcomes. Increasing awareness of CHANTER syndrome's clinical presentation and imaging appearance among radiologists and clinicians is essential for accurate diagnosis and early management.
